# Health Impacts of Chronic Radiation Exposure in Northern Kazakhstan: A Comprehensive Epidemiological Review

**DOI:** 10.3390/cancers18091404

**Published:** 2026-04-28

**Authors:** Polat Kazymbet, Kuralay Ilbekova, Elena Saifulina, Mulkat Yelshenbek, Yerlan Kashkinbayev, Danara Ibrayeva, Moldir Aumalikova, Dinara Bizhanova, Yerbol Dogalbayev, Meirat Bakhtin

**Affiliations:** 1 Scientific Research Institute of Radiobiology and Radiation Protection, NJSC “Astana Medical University”, Astana 010000, Kazakhstan; p.kazymbet@mail.ru (P.K.); saifulina.e@amu.kz (E.S.); yelshenbek.m@amu.kz (M.Y.); kashkinbaev@amu.kz (Y.K.); aumalikova.m@amu.kz (M.A.); bizhanova.d@amu.kz (D.B.); bakhtin.m@amu.kz (M.B.); 2 Department of Interventional Radiology, CF University Medical Center, Astana 010000, Kazakhstan; yerbol.do@umc.org.kz

**Keywords:** chronic radiation syndrome, cancer risk, radon exposure, environmental health, former uranium mining sites

## Abstract

Chronic exposure to ionizing radiation from uranium legacy sites remains a significant public health concern in Northern Kazakhstan. This review evaluates epidemiological, clinical, and environmental evidence published between 2000 and 2025, with particular emphasis on studies conducted during 2014–2023 in the Stepnogorsk region among populations residing near former uranium mining sites. Residents were exposed to annual external gamma doses of approximately 1.0–3.5 mSv and radon-related doses of up to 1.2 mSv. Cancer registry analyses revealed 1913 malignancy cases in the exposed group (vs. 358 controls), with digestive (29%) and respiratory (17%) cancers predominating. Early signs of chronic radiation syndrome (CRS) were consistently observed, including olfactory dysfunction, immunosuppression, vestibular disturbances, hematologic anomalies, and elevated chromosomal aberrations (1.3–1.5× baseline). Non-cancer morbidity was also elevated, including hypertension (32% vs. 24%), chronic bronchitis (14% vs. 8%), and reduced forced expiratory volume in one second (FEV_1_; −9.7%), indicating broader systemic effects. These results underscore the need for targeted public health strategies incorporating CRS biomarker screening, cancer surveillance, personal dosimetry, and environmental remediation in uranium-impacted communities.

## Simple Summary

This narrative review aims to summarize current evidence on cancer epidemiology and long-term health outcomes in populations living near uranium tailings in Northern Kazakhstan. Particular attention is given to early biological effects associated with chronic radiation exposure and their potential role in the development of cancer and other chronic diseases. The review highlights the importance of environmental monitoring, medical surveillance, and public health strategies in communities affected by legacy uranium mining.

## 1. Introduction

The current review was prepared based on findings from environmental and health studies (2000–2025) conducted in Northern Kazakhstan, where populations residing in the vicinity of the uranium mining area have experienced prolonged exposure to ionizing radiation [1]. This exposure stems from the accumulation of long-lived radioactive waste products—primarily uranium tailings—stored in open surface repositories due to extensive uranium extraction and chemical processing operations [2]. Although environmental monitoring data and anecdotal health reports have raised concerns about the potential adverse effects of such chronic radiation exposure, comprehensive epidemiological investigations and systematic clinical evaluations of these residential cohorts have remained limited. As a result, there is a critical need to synthesize existing evidence and identify gaps to better understand the long-term health consequences for communities living near contaminated legacy sites.

Kazakhstan holds a central position in the global uranium industry, both as a major producer and as a nation with substantial reserves. It is currently estimated that nearly 20% of the world’s uranium reserves are within Kazakhstan’s borders, making it a strategic player in global nuclear energy supply chains. The country’s uranium production has seen rapid growth in recent decades and is primarily managed by the state-owned enterprise National Atomic Company Kazatomprom JSC, which oversees exploration, extraction, and processing activities across multiple sites [3,4].

However, Kazakhstan’s uranium legacy is marked by significant environmental and health challenges resulting from poorly regulated mining and milling operations during the Soviet era [5]. According to a comprehensive inventory compiled in 1993, there are 127 legacy uranium mining and milling sites scattered across the country. These sites represent a complex mixture of radioactive contamination, heavy metal pollution, and socio-environmental neglect [6].

Geographically, uranium legacy contamination in Kazakhstan can be broadly divided into three major regions: Northern Kazakhstan, Southern and Central Kazakhstan, and Western Kazakhstan.

Northern Kazakhstan, the region hosts 12 major uranium deposits, notably in the Kokshetau area, including mine groups Nos. 1, 3, 8, 9, and 12. Specific sites such as Kosatchinnoe, Shatskoe, Glubinnoe, Agashskoe, Koksorskoe, and Manybaiskoe have been identified as high-risk due to their proximity to residential areas. The Stepnogorsk Mining Chemical Combine (SMCC) also represents a major site of concern, having generated extensive surface tailings with an estimated 81.2 million tonnes of radioactive waste [7,8].

Southern and Central Kazakhstan: This region contains four key legacy sites, including Kurdai, Vostochniy, and Zapadniy in the Zhambyl region, and Karasaiskiy and Ulken-Akzhal in Central Kazakhstan. Collectively, these areas harbor 117.8 million tonnes of waste, much of which remains insufficiently contained and unremediated.

Western Kazakhstan: The most prominent site in this region is the Koshkar-Ata tailings pond, located near Aktau city. It contains an estimated 58.9 million tonnes of radioactive and toxic waste, making it one of the largest single sources of environmental contamination in the region [9].

Despite the scale of these sites, comprehensive environmental remediation and health risk assessments have been slow to advance. SMCC tailing dumps remain uncovered, vulnerable to wind and water erosion, and situated near populated or agricultural areas, raising concerns about long-term exposure to radiation, groundwater contamination, and ecological degradation. Efforts toward remediation, site stabilization, and epidemiological monitoring remain fragmented, with a pressing need for coordinated national and international action [10].

The SMCC tailings facility consists of multiple uncovered waste ponds situated within 5–10 km of residential settlements such as Aqsu, Kvartsitka, Zavodskoy, and Stepnogorsk city. These sites emit low levels of gamma radiation and release radon gas into both indoor and outdoor air. Despite protective zones established under sanitary legislation, many homes and community buildings fall within areas where dose limits are periodically exceeded. Radiation exposure from uranium mining tailings occurs through multiple pathways, including external gamma radiation from contaminated soil and building materials, inhalation of radon and radioactive dust, and ingestion of radionuclides via contaminated water and food. Groundwater contamination associated with uranium mining activities has also been documented in several studies [11,12]. In Stepnogorsk, deterioration of tailings maintenance since the 1990s, such as loss of water cover and increased dust erosion, has amplified these risks, particularly in settlements located within 5–10 km of the waste ponds [13,14].

While extensive studies have documented the health effects of high-dose radiation from acute exposures, such as those following the Chernobyl accident or atomic weapons testing, growing evidence suggests that chronic low-dose radiation exposure may also represent a risk factor for non-communicable diseases, including tumors and vascular disorders [15]. The biological effects of such exposure are often subtle and may develop gradually over years or decades, making early identification both challenging and critically important [16].

A key concept in this context is CRS, first proposed in Russia in the mid-20th century and formalized through longitudinal work on the Techa River population exposed to chronic emissions from the Mayak plutonium facility. CRS is characterized by a constellation of early, reversible functional impairments, such as sensory, immune, and neurovegetative disturbances, that may precede structural organ pathology by many years [17]. These effects occur at cumulative doses far below thresholds historically associated with deterministic outcomes.

In Stepnogorsk, initial measurements conducted in the 2000s revealed elevated background gamma dose rates (0.15–0.30 μSv h^−1^), with localized peaks near tailings exceeding 0.40 μSv h^−1^. Indoor radon concentrations ranged from 313 to 858 Bq m^−3^, exceeding both WHO guidelines and Kazakhstan’s sanitary norms [18,19]. These conditions translate into annual effective doses of 1–3.5 mSv from gamma exposure and up to 1.2 mSv from radon, corresponding to cumulative exposures of 10–20 mSv per decade for long-term residents [20].

Although environmental measurements raised concerns, the health impacts remained undercharacterized until recent epidemiological and clinical studies began to emerge. Between 2001 and 2015, cancer registry data identified 1913 cases of malignant tumors in the exposed cohort, compared with 358 cases in a control group located approximately 90 km from the SMCC site. The control group consisted of residents of Akkol, located approximately 90 km from Stepnogorsk. This comparison population was selected because of its broadly similar lifestyle and socioeconomic conditions, while being outside the radiation-affected zone. Digestive and respiratory system cancers represented the leading diagnoses [21]. Meanwhile, early functional tests revealed olfactory and gustatory impairment, reduced neutrophil activity, vestibular dysfunction, and mild hematologic deviations—findings consistent with early CRS manifestations.

Importantly, these patterns echo those observed in the Techa cohort, reinforcing the biological plausibility of the findings. However, the Stepnogorsk situation presents a unique opportunity to study low-dose environmental exposure in a civilian population, outside the context of occupational radiation work or post-accident scenarios.

Despite these insights, significant gaps remain. Current studies rarely include individual dosimetry, rely on cross-sectional designs, and seldom adjust for important confounders such as smoking, occupational exposures, and socioeconomic status. Furthermore, there is little integration of clinical biomarkers with long-term population surveillance or public health interventions.

This review primarily focuses on evidence from Stepnogorsk and Northern Kazakhstan, with particular emphasis on studies published between 2014 and 2023. At the same time, the broader literature published between 2000 and 2025 was included to situate the local findings within the wider scientific context of chronic environmental radiation exposure. Although Stepnogorsk is the principal case study, it is considered here as a representative model of long-term exposure in uranium-affected communities of Northern Kazakhstan. The review aims to summarize the available evidence on chronic radiation exposure and its health effects, identify early signs of CRS, and outline both research gaps and practical recommendations. By relating the Stepnogorsk findings to established radiation biology and international evidence, we aim to inform national policy and contribute to the broader understanding of chronic low-dose exposure.

## 2. Methods

A narrative literature review was conducted to summarize the available evidence on cancer epidemiology and long-term health outcomes associated with chronic environmental radiation exposure, with a particular focus on Stepnogorsk and Northern Kazakhstan. Literature searches were performed using PubMed, Scopus, Web of Science, and Google Scholar for publications from 2000 to 2025. The search strategy included combinations of the following terms: “uranium mining”, “uranium tailings”, “chronic radiation exposure”, “radon”, “cancer epidemiology”, and “chronic radiation syndrome”. Original research articles, epidemiological studies, cohort investigations, environmental monitoring reports, clinical studies, and relevant review papers published in peer-reviewed sources were considered. In addition to studies directly related to Stepnogorsk and Northern Kazakhstan, selected international publications on chronic radiation exposure in uranium-affected populations were included when they helped interpret the local findings in a broader scientific context. References cited in the initially reviewed articles were also screened for relevance. The literature was reviewed and organized thematically, with emphasis on cancer epidemiology, early biological effects of chronic radiation exposure, chronic radiation syndrome, and long-term health outcomes in populations living near uranium legacy sites. This review was conducted as a narrative synthesis rather than a formal systematic review or meta-analysis, as the available studies were heterogeneous in design, exposure assessment, and reported outcomes.

The principal sources included in this narrative review are summarized in Table 1.

## 3. Cancer Epidemiology in Exposed Populations

Over the past two decades, cancer registry data from the Stepnogorsk region have consistently shown elevated incidence rates of malignant tumors among residents living within a 10 km radius of the uranium tailings site. Between 2001 and 2015, a total of 1913 cases of malignant tumors were recorded in the exposed cohort, compared with 358 cases in a matched control group residing approximately 90 km away in the Akkol district, an area selected because of its broadly comparable lifestyle and socioeconomic conditions and its location outside the radiation-affected zone [21]. These findings provide the epidemiological foundation for assessing the effects of chronic low-dose radiation on carcinogenesis in the region.

Analysis of site-specific distribution reveals that cancers of the digestive system and respiratory tract predominate in the exposed population. This pattern is biologically plausible, as inhaled radon progeny are known to affect the bronchial epithelium, while ingested radon can deliver a substantial radiation dose to the stomach, which has been identified as a critical organ for this exposure pathway [22,23].

As shown in Table 2, the largest absolute difference was observed for digestive system cancers (+5.3%). A similar absolute difference was also observed for genitourinary tumors (+3.3%), particularly among men aged 51–70 years. Cancer incidence was highest in adults aged 51–70 years, which is consistent with the biologically plausible latency period for radiation-related malignant tumors. Within this age group, men in the exposed cohort had higher incidence rates than those in the control area. Women showed a comparable pattern, with higher frequencies of breast and female genital cancers in the exposed group [21]. Unless otherwise indicated, the differences shown in Table 2 should be interpreted as descriptive comparisons.

Although cancer incidence among individuals under 35 years was relatively low in both the exposed and control groups, the exposed cohort showed a higher frequency. Recent cancer registry analyses indicate that overall incidence rates in the exposed population peaked around 2012–2015 and have since plateaued or slightly declined. However, the distribution of cancer types remained relatively stable, with digestive cancers consistently comprising 28–29% of cases, respiratory tumors 16–17%, and female genital and genitourinary tumors showing no clear change over time [21]. These persistent patterns are consistent with a sustained exposure-related signal, although the contribution of diagnostic trends and population structure cannot be fully excluded.

Radon and its progeny primarily affect the bronchial and alveolar epithelium, causing localized DNA damage through alpha particle interactions. Similarly, ingested radionuclides and external gamma radiation target rapidly proliferating cells in the digestive tract, increasing the likelihood of genetic mutations. Over time, persistent low-level exposure promotes chronic inflammation and oxidative stress, which further disrupt cellular regulation and create a pro-tumorigenic environment in radiosensitive tissues, supporting a biologically plausible link to increased cancer risk [22,24,26]. These mechanisms are consistent with findings from the Techa River cohort and other long-term follow-up studies of uranium-exposed populations in Russia and Central Asia [17,27].

Although the observed increases in cancer incidence and CRS markers are compelling, several limitations must be acknowledged. The absence of individual dosimetry means that exposure estimates are based on proximity to residence and average environmental dose rates, which may not reflect true individual variability. Additionally, potential confounders—such as smoking habits, alcohol use, occupational exposures, and nutritional status—were not systematically accounted for, possibly influencing health outcomes. An additional important limitation is the incomplete availability of information on major non-radiation risk factors in the underlying studies. Variables such as smoking, alcohol use, occupational exposures, and nutritional status were not systematically or uniformly documented, limiting the ability to assess their potential confounding influence on the reported associations. This is particularly relevant for respiratory outcomes, where radon exposure, cigarette smoking, and occupational dust or silica-related lung damage may each contribute independently or jointly to disease risk. Finally, the relatively small size of the key underlying cohorts and the heterogeneity of the available evidence reduce statistical precision and weaken causal inference.

Population mobility and demographic shifts over the study period may have altered cohort composition, introducing selection bias. Lastly, disparities in healthcare access and diagnostic capacity between exposed and control regions could affect cancer detection rates, complicating direct comparisons.

At the same time, the interpretation of internal exposure pathways remains methodologically complex. In uranium-affected environments, internal exposure, particularly due to radon and its progeny, is likely to play a significant role; however, its quantification is subject to considerable uncertainty. Available studies rely primarily on environmental measurements rather than individual-level monitoring, which limits the precision of dose reconstruction. In addition, estimating tissue-specific absorbed doses from alpha-emitting radionuclides is complicated by variability in inhalation dynamics, particle size distribution, and deposition patterns across organs.

Further uncertainty arises from an incomplete understanding of the relative biological effectiveness of alpha radiation under chronic low-dose conditions and from the difficulty of identifying the most relevant target tissues for specific pathological outcomes. These limitations do not invalidate the observed epidemiological patterns but highlight the need for more refined dosimetric approaches in future studies [21].

## 4. Early Signs of Chronic Radiation Syndrome 

Chronic Radiation Syndrome is a clinically recognized condition that arises from long-term exposure to low-to-moderate doses of ionizing radiation, typically over several years. Unlike acute radiation syndrome, which results from short-term high-dose exposure, CRS manifests through gradual functional impairments that affect multiple physiological systems, often long before irreversible structural pathology emerges (Table 2). The concept was first formalized based on observations in the Techa River cohort and its biological plausibility has been increasingly supported by findings in environmentally exposed populations [17], including Stepnogorsk. In residents of Stepnogorsk and nearby villages (within 5 km of SMCC tailings), studies conducted between 2014 and 2023 documented a consistent pattern of subclinical abnormalities across five key physiological systems: sensory, immune, neurovestibular, hematopoietic, and genetic integrity. Some clinical studies have also reported vascular anomalies such as dolichoarteriopathy [28]. These deviations are observed at cumulative radiation doses of 10–20 mSv per decade, primarily from external gamma exposure and internal radon inhalation.

As shown in Table 3, the sensory testing revealed a marked elevation in olfactory and gustatory thresholds, with exposed residents showing 20–35% poorer detection sensitivity relative to reported control or normative reference values. These findings are consistent with chronic neuroepithelial impairment, potentially induced by radon progeny and low-dose gamma radiation affecting the olfactory bulb and peripheral nerves [29]. Although no formal olfactory tests were conducted among Stepnogorsk residents, reports from uranium workers in Northern Kazakhstan document chronic atrophic rhinopharyngitis in 61% of cases, suggesting potential impairment of olfactory mucosa. Such findings are consistent with the sensory component of early CRS. Similar results were previously reported in studies of the Techa River, indicating the high sensitivity of sensory systems to even modest cumulative doses.

The immune system showed early signs of compromise. Neutrophil phagocytic activity was reduced by approximately 15%, while T-cell subpopulation analysis demonstrated a shift in the CD4^+^/CD8^+^ ratio from the typical ~1.5 to approximately 1.1. These changes suggest altered cellular immunity and are biologically consistent with the suppressive effects of chronic radiation on bone marrow progenitors and lymphoid organs. Notably, no frank immunodeficiency was recorded, but these subclinical shifts may increase susceptibility to infection or carcinogenesis [15].

Neurophysiological assessments showed that 38% of exposed residents displayed mild postural instability on Romberg testing, and 22% showed nonspecific θ-wave slowing on *EEG*, higher than the corresponding reference values reported in the original studies [17,25]. These findings indicate central nervous system (CNS) sensitivity to low-dose radiation, possibly mediated through subtle microvascular or inflammatory changes. Importantly, such findings typically precede overt neurological symptoms and may be reversible. Fatigue, mood instability, and sleep disorders were frequently self-reported in health surveys, suggesting early-stage regulatory dysfunction. While comprehensive endocrine screening was not available, these symptoms align with neurohormonal disruption observed in other chronically exposed populations [17].

Chromosomal aberration testing in peripheral lymphocytes revealed a 1.3–1.5× increase in dicentrics and acentric fragments compared to background levels. These biomarkers are gold-standard indicators of ionizing radiation exposure, and their persistence reflects ongoing genomic instability. Given that, dicentrics are specific to radiation-induced double-strand breaks, their elevation even at ~10–20 mSv cumulative doses underscores the biological significance of chronic exposure in this population [27].

The convergence of multisystem functional changes—many of which are measurable, reproducible, and subclinical—is consistent with early CRS-like changes in the Stepnogorsk population. These indicators appear at dose levels below 50 mSv, challenging traditional deterministic thresholds and suggesting a more nuanced understanding of chronic low-dose radiation effects [17,27].

Moreover, the use of validated functional assays (e.g., Sniffin’ Sticks, flow cytometry, *EEG*) enhances the reliability of these observations. Taken together, the findings suggest biological plausibility, temporal coherence, and internal consistency across systems.

These early functional changes constitute a sensitive biomarker panel for CRS detection. Routine use of olfactory testing, lymphocyte profiling, and chromosomal assays may enable early intervention before irreversible clinical damage occurs. Integrating such screening tools into health surveillance programs in uranium-affected communities could significantly improve detection and risk stratification.

## 5. Long-Term Health Outcomes

In addition to elevated cancer incidence and early functional disturbances characteristic of CRS, long-term residents of the Stepnogorsk region exhibit an increased burden of non-malignant chronic diseases. These include cardiovascular and respiratory pathologies, which may arise from chronic low-dose radiation via mechanisms such as vascular inflammation, oxidative stress, and immune dysregulation.

Population-based studies from 2014 to 2023 compared individuals living within 5 km of SMCC tailings (critical zone) with those residing ~90 km away (control zone). The findings are summarized in Table 4. Unless otherwise indicated, the differences shown in Table 4 should be interpreted as descriptive comparisons.

As shown in Table 4, the prevalence of arterial hypertension among adults aged ≥40 years in the exposed population was higher (32% vs. 24% in controls) [31]. Additionally, flow-mediated dilation (FMD) of the brachial artery—a surrogate marker of endothelial function—was reduced by ~1.5%, crossing the clinical threshold (<7%) used to indicate endothelial dysfunction. These findings are consistent with the hypothesis that low-dose radiation may contribute to vascular aging and impaired nitric oxide-mediated vasodilation [32].

This vascular phenotype mirrors results observed in the Techa cohort, where cumulative doses > 0.3 Gy were linked to increased rates of hypertension, atherosclerosis, and stroke [17,27]. In Stepnogorsk, though cumulative doses are lower (~50–100 mSv), similar trends suggest long-latency cardiovascular radiation effects.

Residents living near the SMCC tailing dumps showed a higher prevalence of chronic bronchitis (14% vs. 8%) and an approximately 10% reduction in FEV_1_, indicating impaired lung function. These respiratory deficits may be related to chronic inflammation and irritation associated with inhaled radon progeny, radioactive dust, and other environmental exposures. The concurrent excess of respiratory cancers (17% vs. 12%) is also consistent with the established respiratory effects of radon exposure reported in uranium-exposed populations [33]. These findings highlight the cumulative impact of prolonged low-dose exposure on pulmonary health.

Mortality data, although limited in granularity, suggest a modest elevation in deaths due to ischemic heart disease (IHD) in the exposed population (rate ratio ≈ 1.15) [34]. Increases in chronic obstructive pulmonary disease (COPD) mortality were also reported. While these outcomes may be influenced by confounding factors such as smoking and healthcare access, their alignment with the observed morbidity profile supports the biological plausibility of a radiation-related contribution.

To strengthen the causal link between chronic radiation exposure and observed health outcomes, long-term follow-up with cause-specific mortality data and standardized death certificate reviews is essential. Several biological mechanisms have been proposed to explain the observed associations, and they are supported to varying degrees by experimental and epidemiological evidence. However, the mechanistic basis remains incomplete, particularly for chronic low-dose exposure under environmental conditions. Ionizing radiation induces oxidative stress, leading to endothelial injury that promotes arterial stiffening and increases the risk of hypertension [31]. In the lungs, radiation damages alveolar epithelial cells and impairs surfactant production, contributing to restricted airflow and obstructive spirometric patterns. Additionally, chronic low-dose exposure disrupts normal tissue repair, promoting progressive fibrosis in radiosensitive organs such as the lungs and the vascular endothelium [26,35]. These mechanisms collectively explain the elevated incidence of cardiovascular and respiratory disease in exposed populations and underscore the long-term systemic risks of environmental radiation.

Addressing these outcomes in tandem with cancer surveillance will ensure a more comprehensive response to radiation-related health risks in uranium-impacted communities. In this context, the concept of CRS, developed from long-term observations in chronically exposed populations such as the Techa River cohort, provides a useful framework for interpreting the multisystem functional changes observed in Stepnogorsk. CRS refers to a pattern of gradually developing neurovegetative, hematologic, immune, and sensory disturbances that may emerge after prolonged radiation exposure and may precede overt structural disease [36]. The congruence in exposure pathways, affected systems, and latency periods supports its applicability beyond occupational settings. Recognizing these patterns early may enable timely public health interventions and improve long-term outcomes in environmentally exposed civilian populations.

Another important limitation is the scarcity of comparable cohorts exposed to uranium under similar environmental conditions. Most well-characterized radiation-exposed populations, such as uranium miners, nuclear industry workers, or post-accident cohorts, differ substantially in terms of exposure pathways, dose rates, and population characteristics. In contrast, the Stepnogorsk population represents chronic, low-dose exposure in a civilian setting with combined internal and external pathways, which is less frequently documented. As a result, direct comparisons between cohorts should be interpreted with caution, and parallels drawn with other studies are intended primarily to provide contextual support rather than strict equivalence. For instance, the elevated incidence of respiratory cancers and chronic bronchitis in Stepnogorsk closely parallels findings in uranium miners from the United States, Canada, and Central Asia, where inhalation of radon progeny has been robustly linked to lung cancer through multiple large cohort studies [37]. Although these miners were subjected to occupational exposures quantified in Working Level Months (WLM), the chronic, low-level radon inhalation experienced by Stepnogorsk residents—estimated at 1–1.2 mSv annually—appears to produce similar respiratory pathologies, reinforcing radon’s role as a potent environmental carcinogen. Depleted uranium has been shown to affect human health, particularly the kidneys and central nervous system [38]. Similarly, the vascular and immunological impairments reported in Stepnogorsk populations—including elevated hypertension prevalence, endothelial dysfunction, and reduced neutrophil activity—echo the findings from Chernobyl cleanup workers (liquidators), who received average doses of 50–250 mSv and later demonstrated increased rates of cardiovascular disease, neurocognitive decline, and immune dysregulation [39]. Chronic exposure to uranium has also shown neurotoxic potential, especially in cholinergic pathways [40]. Importantly, while the Chernobyl exposures were acute or subacute and occurred in occupational contexts, the Stepnogorsk scenario involves civilian populations under sustained environmental exposure over decades, highlighting a public health risk that is more insidious and less studied. The case of the Mayak Production Association workers, who received cumulative doses up to 4 Gy, further supports this interpretation. Among Mayak workers, significant excesses in solid cancers, particularly of the lungs and liver, as well as systemic signs of chronic radiation damage, have been documented, despite the difference in dose scale [41]. The similarities in multisystem effects—sensory, immune, hematologic, and genotoxic—even at lower cumulative exposures in Stepnogorsk (~10–20 mSv per decade) underscore the biological plausibility of long-term health deterioration under chronic low-dose radiation. These parallels with higher-dose, well-characterized cohorts from occupational and post-accident settings reinforce the credibility of the Stepnogorsk findings and argue for urgent biomonitoring and policy intervention in similarly exposed civilian populations.

Although several biologically plausible mechanisms have been proposed to explain the effects of chronic low-dose radiation exposure, including oxidative stress, chronic inflammation, endothelial dysfunction, and genomic instability, their integration into consistent mechanistic models remains incomplete. In particular, the pathways linking long-term low-level exposure to CRS-like manifestations remain incompletely established. This limitation complicates the interpretation of epidemiological findings and introduces uncertainty in causal inference.

At the same time, the absence of fully developed mechanistic frameworks reflects the complexity of chronic environmental exposure rather than the lack of biological effect. The convergence of epidemiological observations with emerging clinical and experimental data supports the plausibility of the reported associations. Nevertheless, further research is required to clarify underlying mechanisms, identify critical target systems, and strengthen the biological basis for interpreting long-term health outcomes in exposed populations [42,43,44].

## 6. Conclusions

This review highlights a consistent pattern of adverse health effects among residents living near the SMCC uranium tailings in Northern Kazakhstan. The available evidence indicates an increased burden of both malignant and non-malignant tumors, together with early multisystem functional changes compatible with chronic radiation syndrome-like manifestations. Taken together, these findings support the view that long-term environmental exposure in uranium-affected communities may contribute to clinically relevant health deterioration and therefore warrant continued scientific and public health attention.

The Stepnogorsk findings are important not only as a local case study, but also as a representative example of the broader challenges associated with chronic low-dose radiation exposure in populations living near former uranium mining sites. The convergence of environmental, epidemiological, and clinical observations underscores the need for enhanced biomonitoring, long-term health surveillance, and site-specific environmental remediation. Future research should focus on individual dose reconstruction, longitudinal cohort follow-up, biomarker-based monitoring, and improved assessment of major confounding factors. Public health priorities include targeted cancer and chronic disease surveillance, reduction of radon exposure, strengthening of community risk communication, and the development of prevention and remediation strategies tailored to uranium-affected communities.

## Figures and Tables

**Table 1:** Summary of the principal sources included in this narrative review.

Setting/Region	Source Type	Population	Main Focus	Key Findings Relevant to the Review	Reference
Stepnogorsk area, Northern Kazakhstan	Environmental/dosimetric study	Residents of settlements near uranium-affected areas	Radiation exposure assessment	Reported elevated environmental radiation exposure in settlements near Stepnogorsk, supporting the exposure background for the reviewed health outcomes	[1]
Kazakhstan	Narrative review	Populations exposed to radon and uranium	Health effects of radon and uranium	Summarized evidence linking chronic radon and uranium exposure with respiratory, oncological, immune, and systemic health effects	[15]
Techa riverside settlements, Russia	Clinical/epidemiological observational study	Chronically exposed residents	Early signs of chronic radiation syndrome	Described early multisystem functional changes, including sensory, immune, hematologic, and neurovegetative disturbances compatible with early CRS	[17]
Stepnogorsk, Aqsu, Kvartsitka, Zavodskoy vs. Akkol control area	Population-based epidemiological study	Exposed and control groups	Cancer incidence	Identified 1913 cancer cases in the exposed group versus 358 in the control group; digestive and respiratory malignant tumors predominated	[21]
Finland	Case–cohort study	Residents using drilled well water	Radon in drinking water and stomach cancer risk	Provided contextual evidence on the role of ingested radon and stomach dose in relation to digestive cancer risk	[22]
International	Review article	Radon-exposed populations	Lung cancer risk from radon	Summarized genetic mutations and protein biomarkers associated with radon-induced carcinogenesis; the relationship between radon exposure and lung cancer risk	[23]
International	Epidemiological review	Environmentally and occupationally exposed populations	Non-pulmonary neoplasm risk	Reviewed epidemiological evidence for possible associations between radon exposure and non-pulmonary cancers	[24]
Mayak Production Association, Russia	Registry-based cohort study	Occupationally exposed workers	Chronic radiation syndrome registry	Provided a structured long-term framework for CRS and its multisystem manifestations in chronically exposed populations	[25]

**Table 2:** Distribution of malignant tumors by localization (2001–2015).

Cancer Localization	Exposed Zone (n = 1913)	Control Zone (n = 358)	% Difference (Absolute)
Digestive organs (C15–C26)	560 (29.3%)	86 (24.0%)	+5.3%
Respiratory & mediastinal organs (C30–C39)	320 (16.7%)	56 (15.6%)	+1.1%
Genitourinary system (C60–C63)	212 (11.1%)	28 (7.8%)	+3.3%
Female genital organs (C51–C58)	96 (5.0%)	14 (3.9%)	+1.1%
Breast (C50)	94 (4.9%)	14 (3.9%)	+1.0%
Hematopoietic & lymphoid tissue (C81–C96)	117 (6.1%)	24 (6.7%)	−0.6%
Other/unspecified	514 (26.9%)	136 (38.0%)	−11.1%

Source: adapted from Ilbekova et al. [21].

**Table 3:** Functional markers of early CRS in the Stepnogorsk exposed population.

System	Biomarker/Test	Observed Change (Exposed)	Control Value	Reference
Sensory	Olfactory threshold (Sniffin’ Sticks); Gustatory strips	↑ Threshold by 20–35%	Normative range	[18]
Immune	Neutrophil phagocytic index; CD4^+^/CD8^+^ ratio	↓ Phagocytosis—15%; CD4^+^/CD8^+^ ratio ~1.1 (vs. 1.5 norm)	Normal range	[18]
Neurovestibular	Romberg sway test; *EEG*	Sway in 38% of adults; θ-wave slowing in 22%	<10%; normal EEG	[17]
Hematopoietic	Reticulocyte count; Lymphocyte count	Mild ↓ in reticulocytes; mild lymphopenia	Within normal range	[18]
Genotoxicity	Chromosomal aberrations in lymphocytes (dicentrics, fragments)	↑ Frequency by 1.3–1.5× baseline	0.8‰ ± 0.1‰	[30]

Population: adults residing ≥ 30 years within 5 km of SMCC tailings (critical zone). Table 3 summarizes descriptive differences reported in the literature; formal statistical testing was not uniformly available for all markers. ↑—indicates an increase compared with the control or reference value; ↓—indicates a decrease compared with the control or reference value.

**Table 4:** Prevalence of chronic diseases and physiological impairments in exposed vs. control group.

Health Indicator	Exposed Zone (≤5 km)	Control Zone (~90 km)	Difference	Reference
Arterial hypertension (≥40 y)	32%	24%	+8 pp	[18]
Flow-mediated dilation (FMD), %	6.3 ± 1.1%	7.8 ± 1.0%	–1.5%	[18]
Chronic bronchitis prevalence	14%	8%	+6 pp	[21]
Forced expiratory volume (FEV_1_, liters)	2.78 ± 0.55	3.08 ± 0.50	−9.7%	[18]
Respiratory system tumors ((C30–C39)	17% of all cancers	12% of all cancers	+5 pp	[21]
Mortality from ischemic heart disease (IHD)	Elevated (rate ratio ≈ 1.15)	Reference baseline	-	[21]

## Data Availability

No new data were created or analyzed in this study. Data sharing is not applicable to this article.

## Abbreviations

The following abbreviations are used in this manuscript:

CRS	chronic radiation syndrome
SMCC	Stepnogorsk Mining and Chemical Combine
FMD	flow-mediated dilation
FEV_1_	forced expiratory volume in one second
EEG	electroencephalography
CNS	central nervous system
IHD	ischemic heart disease
COPD	chronic obstructive pulmonary disease
